# The Impact of the Animal Housing System on Immune Cell Composition and Function in the Blood of Dromedary Camels

**DOI:** 10.3390/ani12030317

**Published:** 2022-01-28

**Authors:** Jamal Hussen, Mohammed Ali Al-Sukruwah

**Affiliations:** Department of Microbiology, College of Veterinary Medicine, King Faisal University, Al-Ahsa 31982, Saudi Arabia; 219028688@student.kfu.edu.sa

**Keywords:** housing, immunity, dromedary camel, excitement, flow cytometry, leukocytes ROS, phagocytosis

## Abstract

**Simple Summary:**

The present study investigated the impacts of a change in animal housing system on selected parameters of the camel immune system. Samples collected from camels during a free-ranging time were compared with samples collected from the same camels during movement-restricted housing. Movement-restricted camels showed elevated myeloperoxidase activity in their serum, a significant shape-change of their neutrophils, and higher reactive oxygen species content in their monocytes and neutrophils. The leukogram pattern of the camels under restricted housing was characterized by increased numbers of neutrophils, eosinophils, lymphocytes, and monocytes. Within the lymphocyte population, only the helper T cells and B cells were expanded in animals under restricted housing. In addition, restricted housing modulated the expression of several cell surface antigens, including monocyte-polarization markers and cell adhesion molecules. Functional analysis of bacterial phagocytosis indicated impaired antibacterial function of phagocytes in camels under restricted housing. In summary, the present study identified significant changes in blood immune cell composition, phenotype, and function in dromedary camels under restricted-housing conditions, and suggests the development of an excitement leukogram in those animals.

**Abstract:**

Background: The dromedary camel (*Camelus dromedarius*) is an important livestock animal of desert and semi-desert ecosystems. In recent years, several elements of the camel immune system have been characterized. Stress and excitement induced by animal housing represent the most important environmental factors with potential modulatory effects on the immune system. The present study evaluated the impacts of a restricted-housing system on some phenotypic and functional properties of blood leukocytes in dromedary camels. Methods: Immunofluorescence and flow cytometry were used to comparatively analyze samples collected from camels during a free-ranging time and samples collected from the same camels during movement-restricted housing. Results: In comparison to blood samples collected from the camels during the free-ranging time, samples from movement-restricted camels showed elevated serum myeloperoxidase activity, a significant shape-change in their neutrophils, and higher reactive oxygen species content in their monocytes and neutrophils, indicating increased cellular oxidative stress under movement-restricted housing. The leukogram pattern of the camels under restricted housing was characterized by leukocytosis with increased numbers of neutrophils, eosinophils, lymphocytes, and monocytes, resembling an excitement leukogram pattern. Within the lymphocyte population, only the helper T cells and B cells were expanded in animals under restricted housing. The upregulation of CD163 together with the downregulation of MHC-II on monocytes from excited camels indicate a modulatory potential of animal excitement to polarize monocytes toward an anti-inflammatory phenotype. Functional analysis of bacterial phagocytosis indicates an impaired antibacterial function of phagocytes in excited camels. The downregulation of several cell adhesion molecules on leukocytes from excited camels suggests a role for impaired cell adhesion and tissue migration and leukocyte retention in blood in the observed leukocytosis in animals under excitement. Conclusions: The present study identified significant changes in blood immune cell composition, phenotype, and function in dromedary camels under restricted-housing conditions. The observed changes in leukocyte composition suggest the development of an excitement leukogram pattern in camels under movement-restricted housing. To evaluate the clinical relevance of the observed changes in immune cell phenotype and function for the immune competence of camels under restricted housing, further studies are required.

## 1. Introduction

The dromedary camel (*Camelus dromedarius*) is one of the most important livestock animals in arid and semi-arid regions [1,2,3,4]. In comparison to other domestic species, camels can reproduce and produce high-quality food under extremely harsh conditions, including heat stress and very limited food and water resources [5,6,7]. In addition, camels show relatively higher resistance to several pathogens than many other farm animal species [8].

For the dromedary camel, several components of the immune system have been recently characterized [8]. For most immunological studies, peripheral blood leukocytes, which are a valuable source of innate and adaptive immune cells, are collected from the animals for ex vivo analysis of their phenotypes and functions. The leukocyte count in peripheral blood is regulated by the balance between their production in the bone marrow and their migratory activity from blood to tissues [9]. Leukocyte adhesion to blood vessel endothelial cells and their transmigration and extravasation from blood are regulated by several cell surface adhesion molecules [10,11,12,13].

The phenotypes and functions of leukocytes are influenced by several physiological [14] and pathological factors [15]. Stress and excitement during animal housing represent major environmental factors with significant modulatory effects on the immune system [16]. Studies in several animal species have shown that measurements of many immunological parameters in samples taken from stressed animals can be highly variable, ranging from almost normal baseline to extremely high [16,17,18,19,20]. Cattle and sheep under short-term stress have shown impaired immune responses [17,20]. Bovine heifers with higher handling-stress responsiveness showed reduced antibody and cellular immune responses [16].

Animal stress may result from several factors, which may differently affect animal welfare. Animal stressors may be either physiological, including excitement, restraint, novelty, or handling; or physical stressors, such as hunger, fatigue, injury, or heat-stress [21,22]. According to recent reports, housing management, including space allowance and the freedom of movement, are critical factors for camel welfare [23,24,25]. Restricted housing agitates and excites animals, resulting in changes in several body systems, including the immune system [17,22,26]. In cattle, a limited space allowance has been linked to higher aggressiveness and reduced immune responses [27,28].

The methods of camel housing are now changing due to the increased cultivation, resulting in a significant decrease in free grazing areas. Camel keepers of free-grazing management systems are currently looking for alternative intensive and semi-intensive housing systems [29]. In semi-intensive keeping systems, camels are set free during the daytime and collected at the evening to be herded in closed pen during night. The limited space in intensive animal housing systems is usually associated with several environmental stressors that could affect their welfare [29]. As the impact of housing system on the camel immune system has not been investigated so far, the present study aimed at the evaluation of leukocyte composition, phenotype, and function in camels after movement-restricted housing.

## 2. Materials and Methods

### 2.1. Animals and Blood Sampling

Nine apparently healthy female dromedary camels were selected from a herd of 25 camels reared at a private farm in the Al-Ahsa region in Saudi Arabia. The experimental camels were from the black Majahim breed ranging in age between 9 and 13 years. The she-camels were non-pregnant and non-lactating animals with an average body weight of 345 ± 12.0 kg. As the studied camels were from an associated homogenous herd, the selection criteria were based on the animal age and weight. The study was performed in the arid area of the Al-Ahsa region (N 25°17′8.0844″, E 49°29′11.3316″) in the eastern province of Saudi Arabia. The annual temperature of the area varies from 15 to 45 °C with a mean annual rainfall of 85 mm. The study was conducted on two consecutive days in November 2020 with an average ambient temperature and relative humidity of 23 °C (range: 19–28 °C) and 31.3% (range: 20–32.5%), respectively. The animals were kept under a traditional management system and were not within the breeding season. During the daytime, the camels were grazing, browsing from 06:00 a.m. to 08:00 p.m. In the evening, the animals were guided by the caretaker on foot into the farm to be corralled in a group-field fence during the nighttime (08:00 p.m. to 06:00 a.m.). The camels were fed on hay and barley in addition to bread and discarded dates with a mineral supplement (copper sulphate, zinc sulphate, manganese sulphate, calcium iodine, cobalt sulphate, and sodium selenite). Drinking water was available for the camels in the stall and the grazing range. For the first sampling, blood samples were collected from the free-ranging animals during the daytime (at 09:00 a.m. to 12:00 p.m.), where the animals were distanced from each other (1–3 km distance). The second blood sample was taken from the same camels on the second day at 05:00 a.m., where the animals were still on the farm. Blood samples were obtained by venipuncture of the jugular vein (vena jugularis externa) into vacutainer tubes containing ethylenediaminetetraacetic acid (EDTA). After collection, blood samples were transported to the testing laboratory in a cool box. Cell separation from the collected blood samples was performed within one hour from the sampling. For blood serum collection, blood was taken into sterile tubes without anticoagulants (Becton Dickinson, Heidelberg, Germany). After clotting, cell-free serum was prepared by centrifugation of the blood samples at 3000× *g* for 15 min.

### 2.2. Serum Myeloperoxidase Activity

Serum myeloperoxidase activity was estimated based on the procedure described previously [30,31] with some modifications. Briefly, the test was performed in a flat-bottomed 96-well microtiter plate. Serum was diluted 1 to 5 in phosphate buffered saline (PBS) and 50 μL of diluted serum was incubated with 50 μL of peroxidase substrate and chromogen buffer (33.3 mmol/L citric acid, 66.7 mmol/L NaH_2_PO_4_, pH 5.0, supplemented with 130 µg/mL 3,3′,5,5′-tetramethylbenzidine and 0.01% (*v*/*v*) H_2_O_2_; all chemicals from Sigma, Darmstadt, Germany). After a short incubation of 8–10 min at RT in the dark, the reaction was stopped by adding 50 μL of 0.5 M H_2_SO_4_ to each well. The density of color developed was measured spectrophotometrically (MR 5000, Dynatech, Denkendorf, Germany) at 450 nm.

### 2.3. Cell Separation

Camel white blood cells were isolated from EDTA blood samples after removing the red blood cells using osmotic hypotonic lysis. For this, 1 mL blood sample was incubated in 5 mL distilled water for 20 s, and 5 mL double concentrated PBS were added to restore tonicity. This procedure was repeated until complete hemolysis. After that, the cells were washed two times in PBS (500× *g*, 250× *g*, 10 min, 10 °C). Separated leukocytes were finally suspended in staining buffer (PBS containing 5 g/l BSA, 100 mg/l NaN_3_) at 5 × 10^6^ cells/mL for flow cytometry.

### 2.4. Analysis of Neutrophils’ Shape-Change

For the analysis of changes in neutrophil side scatter characteristic (SSC), which is indicative of neutrophil degranulation and secretion of their granular contents [32,33], separated leukocytes (5 × 10^6^ cells/mL) were analyzed for their mean SSC values using the Accurie C6 flow cytometer (equipped with a blue (488 nm) and a red (640 nm) laser, two light scatter detectors (FSC and SSC), and four fluorescence detectors; BD Biosciences, Heidelberg, Germany). Neutrophil SSC height (SSC-H) was measured using the CFlow^®^ software (version 1.0.264.21, BD Biosciences, Heidelberg, Germany) and compared between cells from normal range animals and animals during restricted housing.

### 2.5. Measurement of Reactive Oxygen Species Production in Neutrophils and Monocytes

For monocyte identification, separated leukocytes were firstly labeled with anti-CD14 antibody. For this, 100 µL per well of 1 × 10^6^ leukocyte suspension were incubated with an APC-conjugated mouse IgG2a against human CD14. After 15 min at 4 °C, the cells were washed twice with MIF buffer. Labeled leukocytes were then incubated (20 min; 37 °C, 5% CO_2_) with 500 ng/mL dihydrorhodamine-123 (DHR-123, Mobitec, Goettingen, Germany). After that, the cells were washed once with PBS (300× *g* for 3 min) and the median fluorescence intensity of FL1 (indicative for ROS amount) was determined by flow cytometry (Accurie C6 flow cytometer, BD Biosciences, Heidelberg, Germany). The fluorochromes allophycocyanin (APC) and DHR-123 were excited by the red and the blue lasers and detected in FL-1 and FL-4, respectively.

### 2.6. Monoclonal Antibodies

The antibodies used for cell staining are presented in Supplementary Table S1. All monoclonal antibodies were directed against leukocyte antigens of other animals, including lama (CD44 and CD45R), human (CD14 and CD18), bovine (CD14, CD163, CD4, WC1, CD11a), and swine (MH II). All antibodies were tested for reactivity against camel leukocytes in previous studies. The cross-reactivity was based on the expression pattern in flow cytometry [15,34,35,36,37]. For the CD4 and WC1 antibodies, a cross-reactivity with camel leukocytes antigens has been indicated by the manufacturer (Supplementary Table S1).

### 2.7. Membrane Immunofluorescence

Cell labeling was performed in a round-bottomed 96-well microtiter plate using 5 × 10^5^ leukocytes per well as previously described [38]. All incubation and centrifugation steps were performed at 4 °C. Separated leukocytes were incubated with unlabeled primary monoclonal antibodies (mAbs) (Supplementary Table S1) specific for the cell surface molecules CD4, WC-1, CD14, CD163, CD172a, MHC-II, CD11a, CD18, CD44, and CD45R [15,34,35,36,37] for 15 min in the dark. After two washings in staining buffer, the cells were incubated with fluorochrome-labeled anti-mouse IgM, IgG1, and IgG2a secondary antibodies (Invitrogen, Schwerte, Germany) for 15 min in the dark. Parallel setups were incubated only with antibody isotype controls. After two washings, labeled cells were analyzed on an Accurie C6 flow cytometer (BD Biosciences, Heidelberg, Germany) by the acquisition of at least 100,000 total leukocytes. Collected flow cytometric data were analyzed using the CFlow Software (V 1.0.264.21; BD Biosciences, Heidelberg, Germany). Leukocyte count was estimated under a microscope using the Neubauer counting chamber after staining of the blood sample with Türk Solution.

### 2.8. Flow Cytometric Analysis of Bacterial Phagocytosis

For the phagocytosis assay, heat killed *Staphylococcus aureus* (*S. aureus*) bacteria (Pansorbin, Calbiochem, Merck, Nottingham, UK) were labeled with fluoresceinisothiocyanate (FITC, Sigma-Aldrich, St. Louis, MO, USA) according to manufacturer instructions. For monocyte identification, separated leukocytes were firstly labeled with APC-conjugated mouse IgG2a against CD14 as described above. Labeled leukocytes were then incubated in 96 well plates (1 × 105/well) with FITC-labeled *S. aureus* (50 bacteria/cell) for 45 min at 37 °C and 5% CO_2_. After incubation (15 min; 4 °C), the percentage of monocytes and neutrophils with elevated green fluorescence among total cells was calculated after flow cytometric analysis.

### 2.9. Statistical Analyses

For statistical analysis, the values for the two groups were compared using the Prism (GraphPad software version 5, GraphPad Software, San Diego, CA, USA). The Kolmogorov–Smirnov test (with the Dallal–Wilkinson–Lilliefor *p* value) was performed to check the normal distribution of data. For normal-distributed data, the paired student’s *t*-test was used to compare the mean of the two groups. For the data that failed to pass the normality test, the Wilcoxon matched-pairs signed ranks test was used to compare the means. The results for each analyzed parameter are presented graphically as mean ± standard error of the mean (SEM). The *p*-values indicating the significance of the differences between means are presented for each parameter.

## 3. Results

In the present study, the leukogram and some phenotypic and functional properties of leukocytes were analyzed in dromedary camels under restricted housing. Samples from free-ranging camels (range camels) were compared with samples from camels during restricted-housing. During the first sampling, where the camels were free-ranging and distanced from each other, the animals were calm and easy to handle. In contrast to this, the movement-restricted camels on the second sampling day were excited and agitated.

### 3.1. Camels under Movement-Restricted Housing Showed Elevated Serum Peroxidase Activity

**Figure 1:**
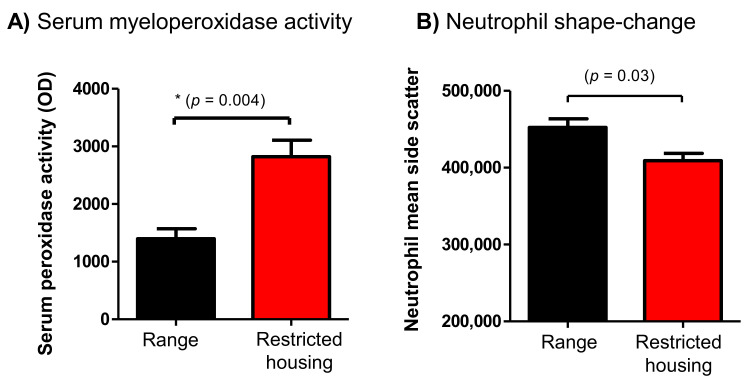
Analysis of serum myeloperoxidase activity and neutrophil shape-change. (**A**) Serum samples were collected from the camels before and after restricted housing and analyzed for myeloperoxidase activity using a colorimetric assay. Optical density (OD) values are presented for free-range and restricted-housing animals as mean ± standard error of the mean (SEM). (**B**) Neutrophil shape-change was analyzed by the assessment of neutrophil side scatter values using flow cytometry. The student’s *t*-test was used to compare the means, and the * *p*-value is indicated on the graph.

The analysis of the enzymatic activity of peroxidase in serum samples collected from range and housed camels revealed significantly (*p* = 0.004) higher activity in housed camels (mean ± SEM; 2822 ± 287 optical density units (OD)) with two times higher values than range camels (1397 ± 175 OD) (Figure 1A). In addition, the SSC values of neutrophils (indicative of granularity) were significantly (*p* = 0.03) reduced in samples collected from housed camels (409,097 ± 9520), when compared to samples collected during the free-range time (452,361 ± 11,230) (Figure 1B).

### 3.2. Camels Leukocytes Showed Higher Spontaneous Production of Reactive Oxygen Species (ROS) during Restricted Housing

**Figure 2:**
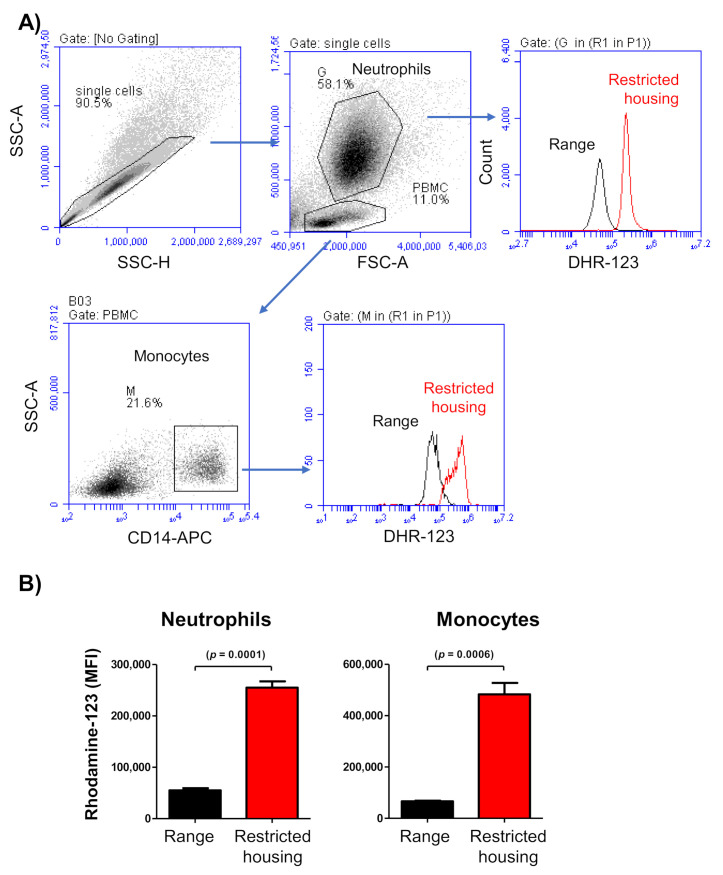
Reactive oxygen species (ROS) levels in monocytes and neutrophils. Leukocytes separated from camel blood were labeled with DHR-123, which detects ROS metabolites. (**A**) Labeled cells were analyzed by flow cytometry. After gating on single cells using SSC-A against SSC-H, neutrophils were identified based on their higher SSC signal. Monocytes were identified as CD14-positive cells within the PBMC population. ROS amount in neutrophils or monocytes was measured as median fluorescence intensity of DHR-123. Cells from free-range and housed camels are compared using an FL-1-histogram. (**B**) ROS MFI values are presented graphically for neutrophils and monocytes (student *t*-test).

The analysis of spontaneously produced ROS by neutrophils and monocytes revealed significantly higher levels in cells from housed camels compared to free-range camels (Figure 2A,B). The MFI values of dehydrorohdamin-123 were three to five-time higher in neutrophils (252,724 ± 12,262; *p* = 0.0001) and monocytes (481,461 ± 44,440; *p* = 0.0006) from housed animals than neutrophils (55,201 ± 4272) and monocytes (66,280 ± 2330) from free-range animals.

### 3.3. Changes in the Leukogram Pattern after Restricted Housing

**Figure 3:**
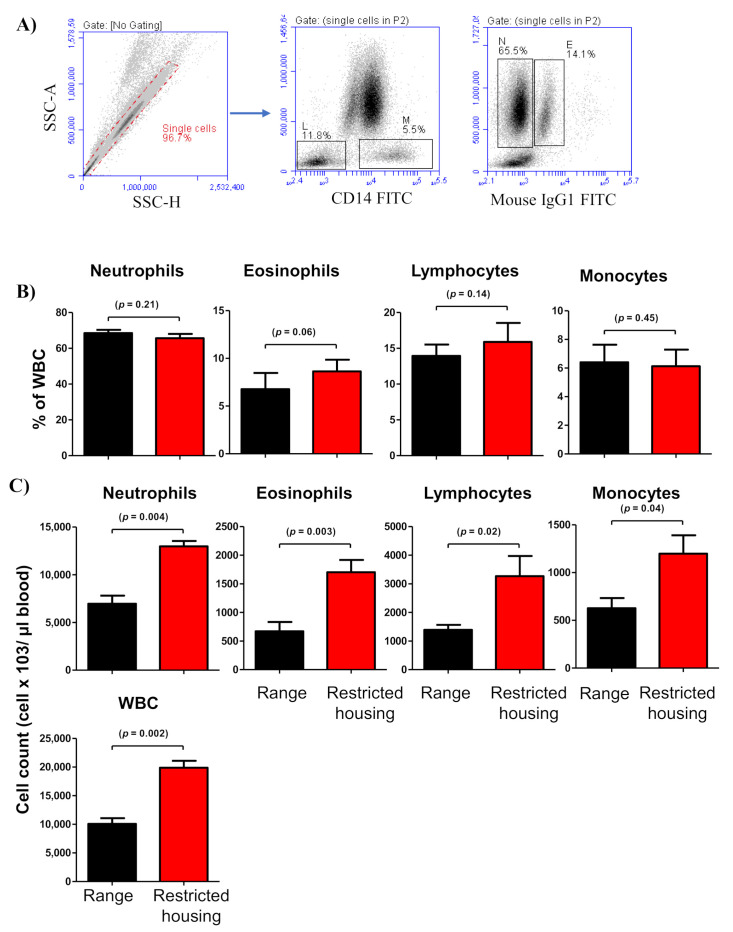
Impacts of housing system on the camel leukogram. (**A**) Gating strategy for the identification of camel leukocyte populations. Singlets were excluded from the analysis based on their side scatter height (SSC-H) and SSC-Aria (SSC-A) signals. Within the mononuclear cell population, lymphocytes (L) and monocytes (M) were identified as CD14-negative and CD14-positive cells, respectively. In an SSC-A/FL-1 dot plot, eosinophils (E) were distinguished from neutrophils (N) based on the higher green autofluorescence of their eosinophilic granules. (**B**) For blood samples collected from free-range and housed camels, the relative percentages (**B**) and the absolute cell numbers (**C**) of all leukocyte populations are presented as mean ± SEM. The *p*-values indicating the significance of the differences between means are presented for each parameter.

Flow cytometric analysis of the relative white blood cell (WBC) composition did not identify any significant differences between housed and free-range camels regarding the percentages of neutrophils (*p* = 0.21), eosinophils (*p* = 0.06), lymphocytes (*p* = 0.14), and monocytes (*p* = 0.45) (Figure 3A,B). The estimations of absolute cell numbers in blood samples from housed and free-range camels revealed significant leukogram changes (Figure 3C). The total leukocyte number in the blood from housed camels (19,880 ± 1214 cell/µL blood) was two-times higher (*p* = 0.002) than in the blood from the free-range camel group (10,080 ± 991 cell/µL blood). The elevated leukocyte number in the blood from housed camels included significantly increased numbers of neutrophils (*p* = 0.004), eosinophils (*p* = 0.003), lymphocytes (*p* = 0.02), and monocytes (*p* = 0.04) (Figure 3C).

### 3.4. Relative and Absolute Quantification of Blood Lymphocyte Subsets

**Figure 4:**
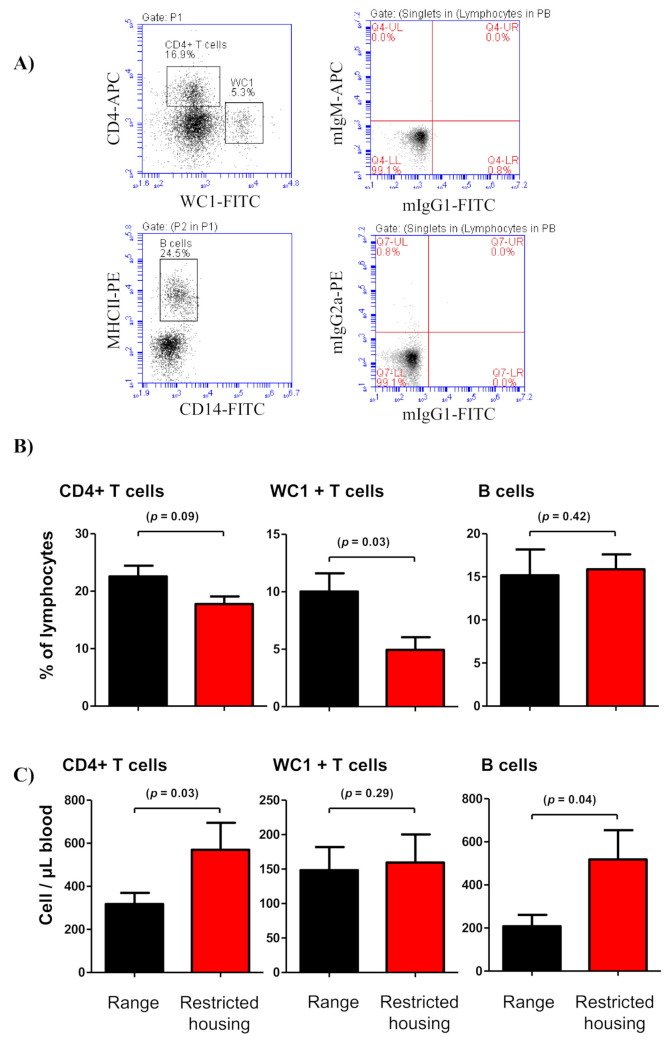
Lymphocyte composition in blood collected from free-range and housed camels. (**A**) Gating strategy for the identification of lymphocyte subsets. The whole lymphocyte population was identified within the mononuclear cells in an SSC-A/FSC-A dot plot and the fraction of CD4-positive T helper cells and WC-1-positive gamma delta (γδ) T cells were identified according to their positive staining with CD4 and WC-1 antibodies, respectively. B lymphocytes were defined based on positive staining with MHC-II molecules and negative staining with CD14 antibodies. The relative percentages (**B**) and the absolute cell numbers (**C**) of B cells, helper T cells, and γδ T cells were estimated and are presented as mean ± SEM. The *p*-values indicating the significance of the differences between means are presented for each parameter.

While the fractions of B cells did not differ between housed and range animals (*p* = 0.42), and the fraction of CD4-positive T helper cells was only slightly (*p* = 0.09) reduced in the blood from housed camels, the percentage of γδ T cells was significantly (*p* = 0.03) lower in the blood from housed camels (Figure 4A,B). In contrast to their relative fractions, the absolute numbers of B cells (*p* = 0.04) and helper T cells (*p* = 0.03) were significantly increased in the blood from housed animals (Figure 4C).

### 3.5. Restricted Housing Modulated the Phenotype of Camel Monocytes

**Figure 5:**
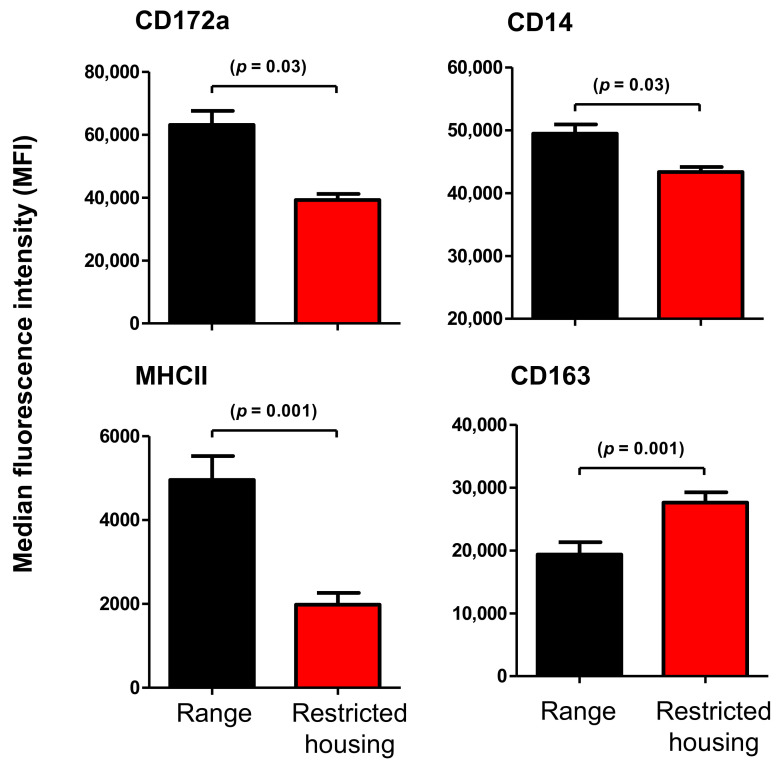
The effect of restricted housing on the expression densities of the main myeloid markers on camel monocytes. Leukocytes were labeled with monoclonal antibodies against CD172a, CD14, MHCII, and CD163, and labeled cells were analyzed by flow cytometry. The abundance of each cell marker was evaluated by median fluorescence intensity (MFI), and the results were presented as mean ± SEM.

Monocytes from housed camels showed a significantly different phenotype in comparison to free-range animals (Figure 5). The median fluorescence intensity (MFI) values of CD172a (*p* = 0.03), CD14 (*p* = 0.03) and major histocompatibility complex (MHC) class II molecules (*p* = 0.001) were lower for monocytes collected from housed camels when compared with those from free-range animals. In contrast to this, the scavenger receptor CD163 was upregulated (*p* = 0.001) on monocytes from housed camels in comparison to those from free-range camels (Figure 5).

### 3.6. Leukocytes from Movement-Restricted Animals Changed Their Adhesion Molecule Expression Profiles

**Figure 6:**
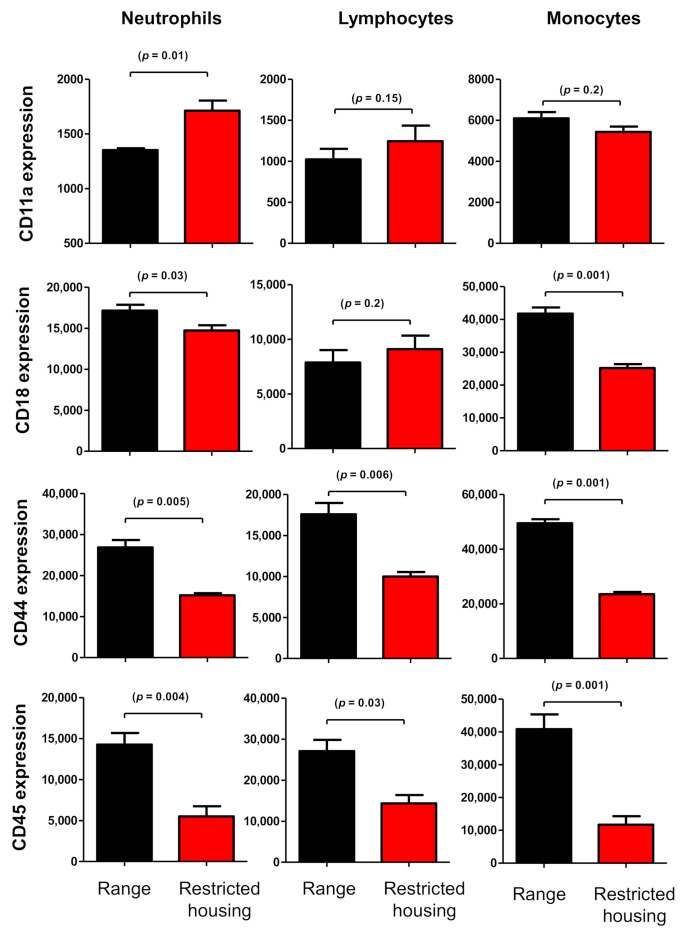
Flow cytometric assessment of the abundances of the adhesion molecules CD11a, CD18, CD44, and CD45 on the surfaces of leukocytes. Leukocytes were labeled with monoclonal antibodies against CD11a, CD18, CD44, and CD45, and labeled cells were analyzed by flow cytometry. After gating on camel neutrophils, lymphocytes, or monocytes, the expression levels of CD11a, CD18, CD44, and CD45 were measured as MFI of analyzed markers, and the results are presented as mean ± SEM (*t*-test).

Restricted animal housing resulted in marked decreases in the abundance of the cell adhesion molecules CD44 and CD45 on all leukocyte populations, including granulocytes, monocytes, and lymphocytes (Figure 6). The average CD44 downregulation was 47.9% (compared to MFI values in free-range animals) for monocytes (*p* = 0.001), 57.9% for granulocytes (*p* = 0.006), and 58.6% for lymphocytes (*p* = 0.009). (Figure 6). Similarly, CD45 was downregulated on granulocytes (38.3% of MFI values in free-range animals; *p* = 0.004), monocytes (28.2%; *p* = 0.001), and lymphocytes (56%; *p* = 0.03) from housed animals in comparison to free-range animals. In addition, housed animals showed increased abundance of CD11a on granulocytes but decreased CD18 expression on monocytes and granulocytes when compared to free-range animals (Figure 6).

### 3.7. The Phagocytic Activity of Monocytes and Neutrophils Was Reduced after Restricted Housing

**Figure 7:**
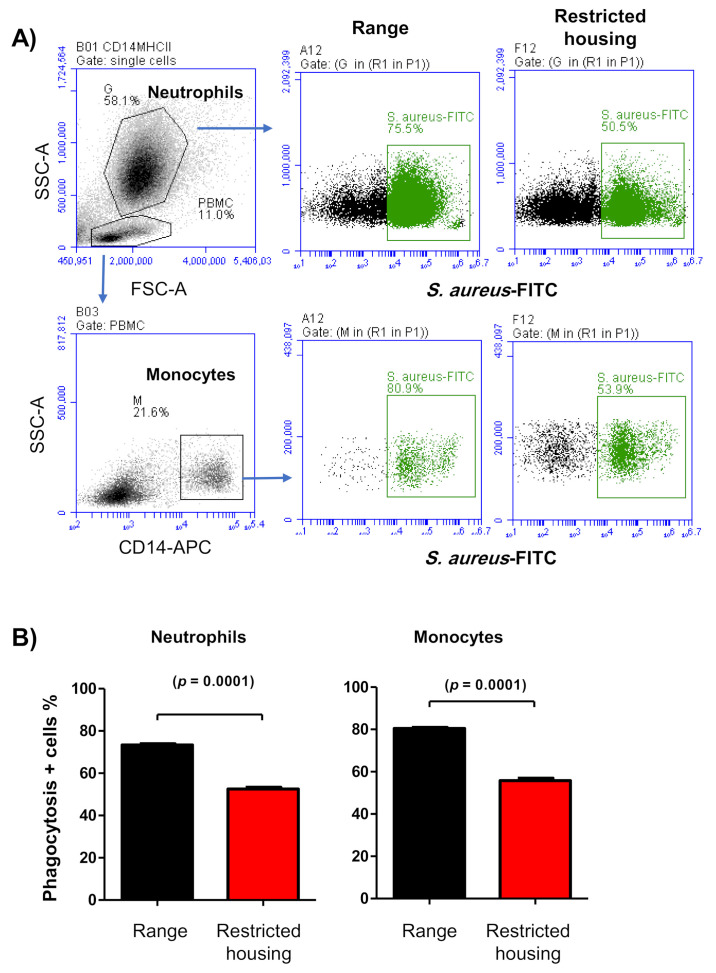
Bacterial phagocytosis by neutrophils and monocytes from free-range and housed camels. Camel leukocytes were labeled with antibodies to the monocyte marker CD14 and were then incubated with *S. aureus* conjugated with FITC. (**A**) Fow cytometric assessment of phagocytosis by neutrophils and monocytes. Neutrophils and monocytes were gated based on their SSC values and CD14 staining, respectively. In an SSC against FL-1 dot plot, the percentage of cells with positive green staining (FITC-*s aureus*) was calculated and presented graphically (**B**) for free-range and housed animals as mean ± SEM (*t*-test).

Flow cytometric evaluation of the capacity of monocytes and neutrophils to uptake *staphylococcus aureus* bacteria revealed a negative effect of restricted housing on the phagocytosis of myeloid cells in camels (Figure 7A,B). In housed animals, the percentages of neutrophils (52.51 ± 1.2% of total cells) and monocytes (55.83 ± 1.1) with ingested bacteria were significantly reduced in comparison to their percentages in free-range animals (73.35 ± 2.1% for neutrophils and 80.48 ± 1.7% for monocytes) (Figure 7A,B).

## 4. Discussion

A functional immune system is essential to maintaining health in animals. The competence of the immune system may, however, be influenced by several physiological and pathological factors. Animal excitement results in changes in several body systems, including the immune system [17,22,26]. According to recent reports, housing management, including space allowance and the freedom of movement, are critical factors for camel welfare [23]. The present study investigated the impacts of movement-restricted housing on selected parameters of the camel immune system.

### 4.1. Increased Cellular Stress in Animals under Restricted Housing

Myeloperoxidase (MPO) represents the most abundant inflammatory enzyme stored in the primary granules of neutrophils, and it can be released upon neutrophil degranulation [39]. Increased serum MPO activity has been identified as an indicator of inflammation and sepsis [40]. MPO, which has strong oxidative activity, catalyzes the formation of reactive oxygen species (ROS). Although ROS produced during oxidative phosphorylation serve as essential regulators of several cellular processes [41], high ROS concentrations in neutrophils may have negative effects on fundamental cellular processes. When released, ROS may also cause damage to various biological structures, such as proteins, carbohydrates, lipids, and nucleic acids, and may enhance inflammatory responses, increasing the risk of tissue damage [42,43]. In the present study, serum samples collected from the camels during restricted housing showed elevated serum MPO enzymatic activity in comparison to samples collected from free-range animals. The induced shape-change of neutrophils—seen through a significant decrease in their mean side scatter values (proportional to cell granularity [32,33,39])—suggests that a restricted-housing system is associated with neutrophil activation and degranulation. These results together with the significantly higher amounts of ROS metabolites in neutrophils and monocytes from housed camels indicate increased cellular stress in animals under restricted housing, which may be associated with enhanced cellular and tissue damage.

### 4.2. Restricted Housing Modulated the Composition, Phenotype, and Function of Camel Leukocytes

Flow cytometric analysis of changes in camel leukogram identified marked leukocytosis in the camels during restricted housing. In other veterinary species, physiologic leukocytosis is usually associated with animal stress, excitation, exercise, or parturition [44]. The typical pattern of physiologic leukocytosis is characterized by neutrophilia and lymphocytosis, which are results of a shift from the marginal leukocyte pool to the circulating pool [44,45]. On the other hand, the corticosteroid-associated leukogram is characterized by lymphopenia and neutrophilia [46,47]. In the present study, the unchanged leukocyte composition together with increased total WBC numbers in housed camels resulted in general leukocytosis with elevated cell numbers of all leukocyte populations, including polymorphonuclear granulocytes (neutrophils and eosinophils) and mononuclear cells (lymphocytes and monocytes). These results argue against a selective effect of restricted housing on the hematopoiesis of distinct immune cell types and suggest the development of an excitement leukogram in camels under restricted housing. However, the analysis of lymphocyte subsets suggests selective increases in only B cells and helper T cells, but not in γδ T cells.

Monocytes are key effector cells of the early immune response to pathogens [48]. Their phenotype is characterized by plasticity, with the ability to differentiate to different functional subtypes of macrophages [49]. The cell surface molecules CD172a, CD14, MHC II, and CD163 are important markers of monocyte phenotype [37,50]. In the present study, monocytes from housed camels displayed a lower abundance of the antigen-presenting molecule MHC II and higher expression of the scavenger receptor CD163, compared to their values before restricted housing, resembling an anti-inflammatory phenotype [48,51,52,53,54,55,56]. This is also supported by the reduced expression of the LPS receptor CD14 on monocytes in addition to their decreased phagocytic activity when compared to cells collected before restricted housing.

We further investigated whether these changes in cell composition and phenotype were associated with functional alterations in the camel immune system. Phagocytosis is an effector innate mechanism with a major role during the first phase of bacterial infection [57,58]. The marked decrease in the ability of neutrophils and monocytes from housed animals to ingest *S. aureus* indicates a negative effect of animal excitement on the innate immune function of camels. The clinical relevance of the observed changes in phagocytic activity, however, requires further clinical studies comparing the in vivo antibacterial capacity of free-range and housed camels.

Leukocyte recruitment to the site of infection and inflammation is an essential part of an effective immune response, which requires their effective interaction with blood vessel endothelial cells and extracellular matrix in a very complex process regulated by the expression of several cell surface adhesion molecules [10,11,12,13]. The hyaluronan receptor CD44 is widely expressed on all leukocyte populations, and it has a crucial role in leukocyte trafficking and inflammatory processes [59,60,61,62]. The lack of CD44 expression was found to be associated with reduced leukocyte recruitment to the site of bacterial infection, which negatively affected the course of infection [63]. The common leukocyte antigen CD45 is a leukocyte cell surface glycoprotein expressed on all cells of the hematopoietic system [64]. CD45 functions as a protein tyrosine phosphatase with a pivotal role in T- and B-cell antigen receptor signaling [64]. It has also an essential role in leukocyte adhesion and migration. The absence of CD45 has been linked to impaired adhesion and migration of macrophages [65]. In the present study, the reduced expression of CD44 and CD45 on all leukocyte populations from the housed camels indicates significant effects of animal excitement due to restricted housing on cell migration and signaling in camel blood leukocytes. The surface molecule CD11a dimerizes with the integrin beta chain-2 CD18 to form the lymphocyte function antigen-1 (LFA-1), a cell adhesion molecule with a major role in leukocyte adhesion and migration [66,67]. The opposite effect of restricted housing on the expression of CD11a and CD18 on granulocytes may argue against a role for LFA-1 in the observed excitement-induced leukocytosis.

The identification of B cells based on their positive staining with MHCII and negative staining with CD14 represents a limitation of the present study, as MHCII is not a cell-specific marker for B cells. However, we tested several monoclonal antibodies against CD20, CD19, IgM, and CD79 of other species for camel leukocytes but did not identify any cross-reactivity with camel B cells (Data not shown). B cells express MHCII, as do other antigen presenting cells, including monocytes and dendritic cells [68]—so using a combination of MHCII and CD14 to exclude monocytes was the only way for us to identify camel B cells. However, as dendritic cells are a minor population in blood, we believe that their contribution to the CD14-MHCII+ lymphocyte compartment would not significantly affect our results. In addition, we found a similar expression pattern using a combination of MHCII and the myeloid marker CD172a, which is also expressed on DC.

## 5. Conclusions

In summary, the present study identified significant changes in blood immune cell composition, phenotype, and function in dromedary camels under restricted housing conditions. Blood samples collected from the camels during movement-restricted housing showed elevated MPO enzymatic activity and higher ROS content in monocytes and neutrophils when compared to samples collected from the same animals during free-ranging time. The leukogram pattern of the camels under restricted housing was characterized by leukocytosis with increased numbers of neutrophils, eosinophils, lymphocytes, and monocytes, resembling an excitement leukogram pattern. Regarding lymphocytes, only the numbers of helper T cells and B cells were affected by restricted housing. In addition, monocytes from excited camels displayed an anti-inflammatory phenotype with elevated CD163 expression and reduced MHC-II expression. Functional analysis of bacterial phagocytosis indicates an impaired antibacterial function of phagocytes in excited camels. The downregulation of several cell adhesion molecules on leukocytes from excited camels suggests roles for impaired cell adhesion, tissue migration, and leukocyte retention in blood in the observed leukocytosis in animals under housing-induced excitement. The evaluation of the clinical relevance of the observed changes in immune cell phenotype and function for the immune competence of camels under restricted housing requires further studies.

## Data Availability

The datasets used and/or analyzed during the current study are available from the corresponding author on reasonable request.

## Supplementary Materials

The following supporting information can be downloaded at: https://www.mdpi.com/article/10.3390/ani12030317/s1, Table S1: List of antibodies.
