# Supplementary material for: The Impact of the Animal Housing System on Immune Cell Composition and Function in the Blood of Dromedary Camels
Source: Animals (Basel). 2022 Jan 28;12(3):317. doi: 10.3390/ani12030317 (PMC8833619; doi:10.3390/ani12030317)
Supplement: Supplementary file 1 [file animals-12-00317-s001.zip › animals-1545236-supplementary.pdf]

animals

MDPI

*Article*

# The Impact of the Animal Housing System on Immune Cell Composition and Function in the Blood of Dromedary Camels

**Jamal Hussen * and Mohammed Ali Al-Sukruwah**

Department of Microbiology, College of Veterinary Medicine, King Faisal University, Al-Ahsa 31982, Saudi Arabia; 219028688@student.kfu.edu.sa
* Correspondence: jhussen@kfu.edu.sa; Tel.: +966-135-896-626

**Citation:** Hussen, J.; Al-Sukruwah, M.A. The Impact of the Animal Housing System on Immune Cell Composition and Function in the Blood of Dromedary Camels. *Animals* **2022**, *12*, 317. https://doi.org/10.3390/ani12030317

Academic Editors: Maria Giovanna Ciliberti and Mariangela Caroprese

Received: 22 December 2021
Accepted: 24 January 2022
Published: 28 January 2022

**Publisher's Note:** MDPI stays neutral with regard to jurisdictional claims in published maps and institutional affiliations.

**Copyright:** © 2022 by the authors. Submitted for possible open access publication under the terms and conditions of the Creative Commons Attribution (CC BY) license (https://creativecommons.org/licenses/by/4.0/).

**Table S1.** List of antibodies.

| Antigen | Antibody clone | Labeling | Source | Isotype |
|---|---|---|---|---|
| CD14 | CAM36A | - | Kingfisher | Mouse IgG1 |
| CD14 | Tuk4 | APC | Thermofisher | Mouse IgG2a |
| MHCII | TH81A5 | - | Kingfisher | Mouse IgG2a |
| CD172a | DH59b | | Kingfisher | Mouse IgG1 |
| CD163 | LND68A | - | Kingfisher | Mouse IgG1 |
| CD4 | GC50A1 | - | Xceltis | Mouse IgM |
| WC1 | BAQ128A | - | Xceltis | Mouse IgG1 |
| CD11a | HUH73A | - | Kingfisher | Mouse IgG1 |
| CD18 | 6.7 | FITC | BD | Mouse IgG2a |
| CD44 | LT41A | - | Kingfisher | Mouse IgG2a |
| CD45 | LT12A | - | Kingfisher | Mouse IgG2a |
| Mouse IgM | poly | APC | Thermofisher | Goat IgG |
| Mouse IgG1 | poly | FITC | Thermofisher | Goat IgG |
| Mouse IgG2a | poly | PE | Thermofisher | Goat IgG |

MHC: Major Histocompatibility Complex; WC1: workshopcluster 1; APC: Allophycocyanin; FITC: Fluorescein isothiocyanate; PE: Phycoerythrin; poly: polyclonal.
